# Key considerations when choosing a retinal camera for diabetic retinopathy screening

**Published:** 2023-07-07

**Authors:** Covadonga Bascaran

**Affiliations:** 1 Clinical Research Fellow, London School of Hygiene and Tropical Medicine, UK.

**Retinal cameras are widely used in DR screening. There are several important aspects to consider when choosing a retinal camera, including how and where it will be used, and the images it is able to produce.**

**Figure 1:**
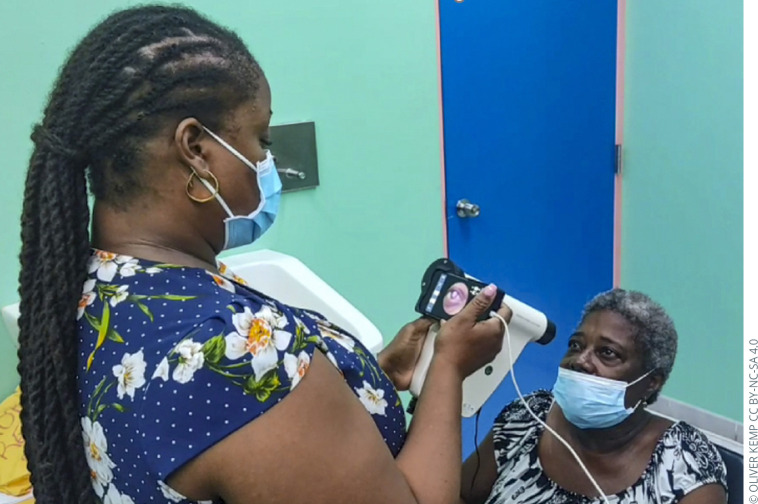
Diabetic retinopathy screening using a hand-held camera. **DOMINICA**

Screening people for diabetic retinopathy (DR) involves examining the retina of patients with diabetes to detect abnormal changes; this is usually done once a year.

The development of retinal cameras has sped up the process significantly. It is now possible to take photos of the retina that can be examined by a specially trained screener/grader, avoiding the need for an ophthalmologist to examine every patient. Thanks to the accuracy and speed of this approach, it has become the standard method for retinal examination in DR screening programmes.^1^ This is particularly helpful due to the growing magnitude of diabetes worldwide, and the shortage of ophthalmologists available.

In recent years, retinal cameras have evolved considerably in both specification and cost. Since health budgets are often overstretched, investing in the right camera for a DR screening programme the first time will save costs later on.

## Table-top or hand-held?

There are two main types of cameras: the traditional static camera which sits on top of a table (also known as a table-top camera), or a hand-held camera (see Table 1).

**Table 1:** Features, advantages, and disadvantages of table-top and hand-held retinal cameras

	Table-top camera	Hand-held camera
**Size and portability**	Large, very difficult to move	Light, easy to move around (can be held in one hand)
**Storage and security**	Must be kept in a dedicated, secure (lockable) area with enough space for patient and operator	Smaller, portable, easy to store, e.g., in a safe or secure, locked cupboard
**Cost**	More expensive	More affordable
**Image quality**	Higher quality images, more consistent quality	Image quality not as good; quality also more variable
**Ease of use (for the operator)**	Easy to use. Has automatic image-capturing capabilities	Manual image capturing; requires more practice to take good quality images that are in focus
**Patient comfort**	Patients have to sit at the table and use the chin rest, which may be uncomfortable for some	Patient can be in any position that is comfortable for them

**Table-top retinal cameras** are large and heavy, and are mounted on a medical instrument table. They must be plugged into a power socket. Patients sit on a chair in front of the camera, with their chin positioned on the chin rest.

**Hand-held retinal cameras** are light, battery-powered cameras that can be used in any position that is comfortable for the patient.

Choosing between the type of camera depends on several factors, as set out below.

### The location where the camera will be used

If the camera is going to be based at a hospital and doesn’t need to be moved, and there is adequate, secure space, a table-top camera could be a good choice.

However, if a programme requires the camera to be used in different clinics, for example when screening at diabetes clinics in primary care facilities, a portable camera would be a better option.

### The financial resources of the programme

Hand-held cameras are relatively inexpensive compared with table-top cameras, and may be a better option when resources are limited and/or when more than one camera is needed.

### The personnel who will be taking the images

Ideally, cameras should be easy to operate by trained non-technical staff. Most table-top cameras come with automatic image-capturing capabilities that make them easy to use with relatively little training. In contrast, some hand-held cameras may require more manual input to achieve good images; therefore, more training and practice is required.^2^

### The image quality

Generally, table-top cameras are more consistent at producing high quality images. When using hand-held cameras, there can be movement of the patient and of the screener, which affects the image focus and therefore the quality of the image. Some hand-held cameras come with an optional portable frame with a chin rest that allows for mounting on a table and makes image acquisition easier.

**To dilate or not?**

Some retinal cameras are marketed as being ‘non-mydriatic’; in other words, images can be taken without the need for pupil dilation. However, even with these cameras, the quality of the images is generally better if pupils are dilated. The decision to dilate or not should be taken depending on the needs of each programme and on local guidelines.^3^

**Figure 2:**
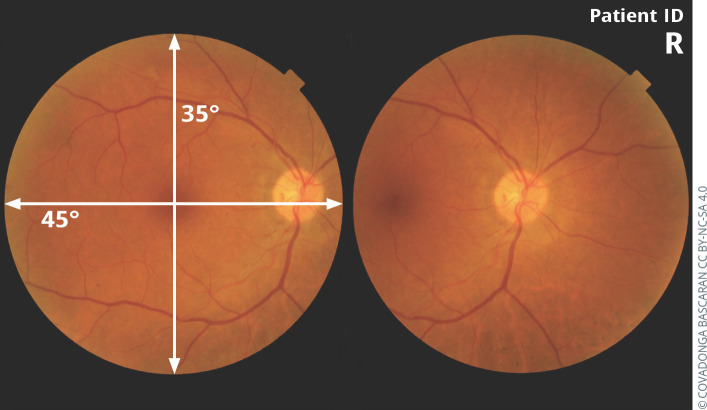
The minimum field of view produced by retinal cameras should be 45° horizontally and 35° vertically. Images should display the patient identification number and a label indicating which eye it is (right or left).

## General considerations

Once you have decided on the most appropriate type of camera for your screening programme and are evaluating different models, consider the following questions.

### Does the camera produce images that meet the minimum specifications?

The images produced by the retinal camera must meet these minimum specifications^4^:

- The minimum field of view should be 45° horizontally and 35° vertically (Figure 2), with a resolution of at least 30 pixels per degree.
- The camera software should be capable of identifying whether the image is of the right or left eye, and label the top right-hand corner of the image accordingly (Figure 2).
- The image colour should be representative of the colour that the operator would see by direct examination of the retina.
- There should be a patient identifier (patient ID) visible on the image; this is one of several standards for retinal imaging^5^ included in the DICOM standard (Figure 2).^6^

### Does the camera itself meet quality standards?

It is important to ensure that the retinal camera complies with international quality standards. Two international standards that many companies seek to comply with are the International Organization for Standardization’s ISO 10940 (2009) standard, and the International Electrotechnical Commission’s IEC 60601-1 standard. In some countries, retinal cameras must also be registered with national or regional bodies.

**“It is important to ensure that the camera complies with international quality standards.”**

### What servicing and support is available locally?

Ensure that the manufacturer gives at least 12 months warranty, and that the parts will be available for at least five years. You should ideally work with a distributor that has a local presence so that they can help with installation, servicing and repairs. If it is likely that the camera will need to be shipped to another country for repairs, you may wish to negotiate with the distributor to provide a camera on loan while repairs are being made, so that the screening programme is not interrupted.
